# Dataset on infant mortality rates in Brazil

**DOI:** 10.1186/s13104-023-06425-9

**Published:** 2023-07-17

**Authors:** Gabriel Souto, Matheus Miloski, Carlos Leonardo Souza Cardoso, Vinicius Kreischer, Balthazar Paixão, Victor de Paula Dornellas Ribeiro, Raquel Gritz, Lucas Carraro, Sérgio Ricardo de Borba Cruz, Carmen Lúcia Corrêa Bonifácio, Raphael de Freitas Saldanha, Leandro Zirondi, Gizelton Pereira Alencar, Ariane Camilo Pinheiro Alves, Nelson Niero Neto, Rebecca Salles, Jefferson da Costa Lima, Marcel Pedroso

**Affiliations:** 1 Platform of Data Science applied to Health (PCDaS), ICICT, Oswaldo Cruz Foundation, Fiocruz, Rio de Janeiro, Brazil; 2 Federal Center for Technological Education of Rio de Janeiro, CEFET/RJ, Rio de Janeiro, Brazil; 3 Data Extreme Lab, National Laboratory for Scientific Computing, DEXL Lab/LNCC, Petrópolis, Brazil; 4 Federal University of Rio de Janeiro, UFRJ, Rio de Janeiro, Brazil; 5 School of Public Health, University of São Paulo, FSP/USP, São Paulo, Brazil; 6 National Laboratory for Scientific Computing, LNCC, Petrópolis, Brazil

**Keywords:** Health informatics, Infant mortality rates, Statistics, Data science

## Abstract

**Objectives:**

Surveillance of infant and fetal deaths is of paramount importance in thinking about government strategies to reduce these rates, provide greater visibility of these mortality figures in the country, enable the adoption of prevention measures, as well as contribute to a better record of deaths.

**Data description:**

The dataset comprises fetal, neonatal, early neonatal, late neonatal, and perinatal Mortality Rates of Brazilian municipalities with their respective information, between 2010 to 2020, aggregated by epidemiological week.

## Objective

Providing strategies to support the epidemiological surveillance of infant and fetal mortality is one of the priorities of the Brazilian Ministry of Health (MS), as it aims to reduce the country’s high mortality rates [1].

Reducing infant mortality is a major challenge for any society’s health services. This is a part of the new 2030 global agenda, Sustainable Development Goals (SDG), specifically in SDG 3.2, which aims to end preventable deaths of newborns and children under five years old. Despite progress, there are still obstacles to overcome in achieving this goal [2].

The Infant Mortality Rate (IMR) is an indicator used to measure infant mortality from the following formula:

$$\begin{aligned} \frac{\text {Number of deaths of children under 1 year of age}}{\text {Number of live births}} X 1.000 \end{aligned}$$

According to the Epidemiological Bulletin of the Secretary of Health Surveillance of the Brazilian Ministry of Health from 1990 to 2019, there was a reduction in IMR in Brazil and in all its Regions. “In 2019, it was estimated that there were 38,619 infant deaths in Brazil, implying an infant death completeness rate of 91.4% and an IMR of 13.3 deaths per thousand live births, returning to the same level as in 2015. The largest reductions were observed in the states of the Northeast Region. In 2019, the lowest and highest IMR were estimated for the Federal District and Amapá, with 8.5 and 22.9 deaths per thousand live births, respectively” [3].

The dataset built by the team of the Platform of Data Science Applied to Health (“Plataforma de Ciência de Dados Aplicada á Saúde” - PCDaS) [4] fulfills its objective, which is to calculate the mortality rates in all Brazilian municipalities, aggregated by epidemiological week. By calculating these mortality rates, it is possible to analyze population and geographic variations in mortality, in addition to contributing to the assessment of health levels and socioeconomic development of the population, in order to support planning processes and political actions aimed at prenatal care, childbirth and the newborn, as explained in [1].

## Data description

The mortality rates (fetal, early neonatal, late neonatal, neonatal, and perinatal) calculated in this work are present in the *mr-mortality-rates* [5] file along with geographic information about the respective Brazilian municipalities, aggregated by epidemiological week.

**Table 1:** Overview of data files/datasets

Label	Name of data file/dataset	File type (file extension)	Data repository and identifier
Dataset	mr-mortality_rates	Delimited text (.csv)	Synapse: https://doi.org/10.7303/syn47199836 [5]
Data file 1	mr-dict	Delimited text (.csv)	Synapse: https://doi.org/10.7303/syn4719361 [6]
Data file 2	mr-municipalities	Delimited text (.csv)	Synapse: https://doi.org/10.7303/syn47194116 [7]
Data file 3	mr-federation_units	Delimited text (.csv)	Synapse: https://doi.org/10.7303/syn47194124 [8]
Data file 4	mr-extract_transform	Jupyter Notebook (.ipynb)	Synapse: https://doi.org/10.7303/syn47028493 [9]
Data file 5	mr-load	Jupyter Notebook (.ipynb)	Synapse: https://doi.org/10.7303/syn47028485 [10]
Data file 6	mr-utils	Python code (.py)	Synapse: https://doi.org/10.7303/syn47199024 [11]

This dataset and related files are available for download as shown in Table 1

### Data construction - methodology

The development of this dataset is the result of a 3-step construction process: (i) extraction of public health data, (ii) data enrichment, and (iii) data validation and integration of annual bases.

i The generated datasets combine data from the Live Birth Information System (SINASC), the Mortality Information System (SIM), and the Mortality Information System Fetal Death Certificate (SIM-DOFET). These datasets are public at DATASUS and represent individual records of live births, deaths, and fetal deaths. The records were aggregated by epidemiological year and week, federative unit, and municipality (mother’s place of residence). The three fundamental datasets were filtered from the epidemiological year 2010. They were also selected in SIM and SIM-DOFET neonatal deaths and then separated into general (0–27 days), early (0–7 days) and late (8–27 days) neonatal deaths, perinatal deaths (22 completed weeks of gestation to seven completed days after birth), and fetal deaths (22 completed weeks of gestation).
ii The filtered datasets from the extraction of the different databases were grouped into a single general dataset containing information on fetal, neonatal, early neonatal, late neonatal, and perinatal mortality. Then, the data was enriched with information about the mother’s municipalities, states, and Brazilian regions of residence, thus generating new attributes in addition to the calculation of mortality rates.
iii The database comprises 38 variables (columns) and 2,659,082 records (rows). Each row is equivalent to a municipality in an epidemiological week of a given year. There are 10 attributes referring to the number of deaths and their respective rates (fetal, early neonatal, late neonatal, neonatal and perinatal), 1 attribute referring to the number of births, 4 carrying temporal information, and 23 referring to spatial information. The values are relative to the places of residence and not the places of occurrence.
iv Since mortality rates were calculated for the lowest possible levels of aggregation, municipalities, and epidemiological week, the researcher can perform aggregations at higher levels in space and time.

## Limitations

- Due to the aggregation by epidemiological weeks and municipalities, multiple records do not have births or deaths and, consequently, do not have their respective values on infant mortality rates. When the record has births but no deaths, the rate is blank. Approximately 0.3% of the records have the births field empty. Around 92% of the records have no fetal mortality rate, 96% have no early neonatal mortality rate, and 98% have no late neonatal rate. Meanwhile, neonatal and perinatal mortality rates are missing in about 95% and 99% of records, respectively. Roughly 88% of the lines have neither rate. The southern region is the most affected, and this occurs in about 92% of its records. The least affected is the northern region, with 84%. The years up to 2015 also do not have a rate in approximately 88% of its records, which rises to about 89% in the following years.
- The infant mortality rate is calculated per thousand births. However, only 1,147 records (approximately 0.04% of the database) have a number of births greater than or equal to one thousand. This occurs relatively uniformly across all regions, states, and years. The Federal District (midwest region) has no record of a number of births greater than one thousand.
- There are 438 records (approximately 0.01% of the dataset) in which the number of deaths exceeds the number of births. The north is the least affected region (only about 0.006% of its records), and the southeast is the most affected one (about 0.02%). The year 2014 is the most affected (approximately 0.022%), and 2018 the least (0.011%). Numerous deaths combined with an excessive number of births lead to high rates.
- The dataset requires correction of the under-enumeration of deaths and live births (the latter on a smaller scale) for directly calculating the rate from data from continuous recording systems, especially in the North and Northeast regions. These circumstances dictate the use of indirect calculations, based on age-proportional mortality, concerning the infant mortality rate estimated by specific demographic methods.

## Data Availability

The data described in this Data Note are freely and openly available on the Synapse repository (https://doi.org/10.7303/syn26343262). The individual data files are available at: mr-mortality_rates [5]; mr-dict [6]; mr-municipalities [7]; mr-federation_units [8]; mr-extract_transform [9]; mr-load [10]; mr-utils [11].

## Abbreviations

MS: Brazilian Ministry of Health, “Ministério da Saúde” in Portuguese.
Fiocruz: Oswaldo Cruz Foundation, “Fundação Oswaldo Cruz” in Portuguese.
PCDaS: Platform of Data Science Applied to Health, “Plataforma de Ciência de Dados aplicada á Saúde” in Portuguese.
SINASC: Live Birth Information System, “Sistema de Informações de Nascidos Vivos” in Portuguese.
SIM: Mortality Information System, “Sistema de Informações sobre Mortalidade” in Portuguese.
SIM-DOFET: Mortality Information System Fetal Death Certificate, “Sistema de Informações sobre Mortalidade - Declaração de óbitos Fetais” in Portuguese.y
